# The influence of gender ratios on academic careers: Combining social networks with tokenism

**DOI:** 10.1371/journal.pone.0207337

**Published:** 2018-11-16

**Authors:** Constantin Schoen, Katja Rost, David Seidl

**Affiliations:** 1 Institute of Sociology, University of Zurich, Zurich, Switzerland; 2 Department of Business Administration, University of Zurich, Zurich, Switzerland; Indiana University Bloomington, UNITED STATES

## Abstract

This paper examines how gender proportions at the workplace affect the extent to which individual networks support the career progress (i.e. time to promotion). Previous studies have argued that men and women benefit from different network structures. However, the empirical evidence about these differences has been contradictory or inconclusive at best. Combining social networks with tokenism, we show in a longitudinal academic study that gender-related differences in the way that networks affect career progress exist only in situations where women are in a token position. Our empirical results further show that women not in severely underrepresented situations benefit from the same network structure as men.

## Introduction

Many researchers who study networks have taken an interest in gender-related differences in the structure of individual professional networks and how they affect the success of their members at the workplace [1]. Some have argued that, compared to their male colleagues, women are more often excluded from professional networks [2, 3] and have fewer professional network contacts [4]. Further, there are arguments that women need different professional networks for career success [5–7], and that they use professional networks less effectively [6–8]. However, the empirical evidence about these differences is contradictory or inconclusive at best. A range of empirical studies that have explored different settings have found no gender-related differences in the configuration and effects of professional networks regarding various outcomes. For example, in their study of bonus payments Gargiulo et al. [9] showed that both women and men benefitted from the same network structures. In a related study that focused on the promotion speed of academics in France, Sabatier [10] showed that the effects of networks were the same for both genders.

In this study, we seek to address this apparent contradiction in the literature. We achieve this by focusing on how the configuration of individual professional networks influences the chances of successful promotion among men and women respectively. More specifically, we draw on social network theory and tokenism theory [5, 11, 12] to argue that gender-related network differences are a result of differences in the proportion of women in the respective work context. To develop our argument, we use the ego network configurations in the professional context [13, 14]. An ego network is a network consisting of the personal contacts of an individual. Burt [5, 13] and others have shown that the number of structural holes in an individual network matter for various outcomes [14–16]. Broadly put, the concept of structural holes describes potential gaps in a social structure if the person who occupies such a hole would not exist. Following Burt’s [1, 5, 13] categorization, we use the number of structural holes in an individual network to distinguish between two types: In the first type a person occupies many structural holes [5]. Such individuals have more opportunities to act as so-called brokers, giving them access to exclusive information and resources. The second type describes individuals that occupy few structural holes. For these individuals the access to exclusive information and resources relies on the support of sponsors who are occupying more structural holes [5].

To date, hardly any studies have examined whether the number of women within an organisation or work unit moderates the influence that professional networks have on career outcomes. In fact, earlier works tended to report the proportions of men and women in the entire organisation (e.g., [13]) or in the study’s sample (e.g., [17]). As we argue, this approach is mistaken, because the proportion of women in a specific context changes the circumstances that influence career advancement [12, 18]. For that reason, in our study we relate specific network configurations to the distribution of men and women in a particular work context. We predict that gender-related differences exist only in contexts where women are extremely under-represented; i.e., where women have what is known as token status [19]. When the share of women is sufficiently high, we expect that such differences disappear. As we will argue, the explanation for this pattern lies in the legitimacy deficit associated with token positions [1, 11]. These legitimacy deficits restrict an individual’s potential to benefit from structural holes. Therefore, we show that individual career outcomes depend on the broader social context (proportion of women).

Based on this argument we develop hypotheses on how gender proportions, network structures and the speed of internal promotion interrelate. To test the hypotheses, we relied on a unique data set of researchers that have reached different professorial ranks at a large university in Switzerland. We generate our network data by matching four types of affiliations: co-authorship, teaching collaborations, university committees and research groups. The context of a large university with different faculties seems particularly suitable for our purpose. Faculties (e.g., the Faculty of Medicine) are units that encompass different research disciplines (such as the Department of Physiology and the Department of Neuroscience) and are responsible for research, teaching, and public services in their respective fields. In other countries, the different units within a university often are termed schools. Different faculties have distinctly defined boundaries and the career paths within each faculty are clearly discernible. Researchers tend to advance their careers in a particular faculty and, as long as they work at the same university, they remain within the same faculty [20]. In Switzerland, the academic system is different to the US or the UK [21]. An internal promotion happens regularly, but is not taken for granted. The fact that there is only a small number of universities in Switzerland and that those universities have a very hierarchical structure creates a tough internal competition for promotions [22]. The result is a long internal career, similar as it is the case in other European countries [20]. This pattern allows us to focus on one organisation and to compare relatively independent sub-organisations, i.e., faculties. Therefore, the career at a Swiss university is similar to an internal labour market and fits into the field of comparative studies.

The contributions of our paper are twofold. First, our paper contributes to the literature on gender differences in professional networks. It can be seen as a response to calls for more research on the claim [1] that women benefit more from networks containing few holes [7]. Taking up this call, we examine gender proportions as a potentially key context variable and highlight the effects of gender ratios on professional networks. In addition, our research contributes to a call from Kossek [23] for more research regarding the different consequences for men and women by using social networks. Second, we contribute to the debate on the sources of gender-related differences in network preferences. We offer evidence for the argument that network differences are not inherently related to gender, as some suggest [6, 8, 24], but are shaped by the context or situation in which they arise [2, 24, 25].

## The prospects of career advancement for women in advanced positions

### The configuration and impact of professional networks

There is a large body of literature on how differences in the structure of professional networks affect career advancement. Professional networks play a key role in career success. For example, they grant access to information and resources, which is particularly important in higher-level jobs [5, 26, 27]. One reason for this argument is that the people who determine who gets promoted tend to favour candidates to whom they have personal relations. This reduces the risk of adverse promotions [28]. A second reason is that advantageous network relationships may help potential candidates gain a better understanding of the relevant evaluation criteria and prepare for their promotion accordingly [28]. A third reason is that having a strong position within a professional network can help foster innovation and creativity and thus increase one’s prospects of promotion [13, 14, 29]. Particularly in the academic context, network relationships can affect both the performance and reputation of researchers [14, 30].

Apart from personal relationships as such, the structural features that characterize a professional network affect career chances. Our study is based on the work of Burt [1] and therefore focuses on the network configuration of a single individual. This individual network is called ego network. While individuals are embedded in larger networks, every individual has a specific network configuration and knows actors that others do not know. This offers individuals the chance to act as broker. Individuals who have many opportunities to act as brokers, in the sense that they bridge otherwise unconnected people, receive more non-redundant—that is, unique—information. Thus, they tend to be better informed about imminent openings or impending disasters [15]. The idea that brokers have certain advantages within their network can be explained by the notion of structural holes. A structural hole is ‘a relationship of non-redundancy between two contacts’ [5]. This means that an individual who occupies the space of a structural hole has the chance to function as an intermediary who facilitates the exchange of information and resources between people who are otherwise unconnected [5]. The Supporting Information file, S1 Fig illustrates the position of a structural hole. Among closely connected peers, the information that circulates is redundant. Whereas the information that the members of a second group have is non-redundant, i.e., unique, from the viewpoint of the first group. Brokers are the ones who connect people from different groups and who have access to non-redundant information that the members of other groups are not aware of. As a result, brokers are in an advantageous position. Individuals who occupy many structural holes (i.e., brokers) tend to be particularly prominent in their organisation and have high chances of promotion [5].

In contrast, individuals that occupy only few structural holes, have severely limited opportunities for brokerage. Typically, those individuals have many connections to direct peers but only few connections to other or distant groups. Accordingly, a way to gain access to exclusive information and resources is to have strategic partners. These partners, so-called sponsors, occupy more structural holes [1, 31]. Sponsors lend the advantages from structural holes to ego. S2 Fig illustrates the lending of structural holes advantages. This lending position of ego creates a dependency. If ego’s connection to a sponsor deteriorates, he or she loses the benefits of the structural holes that are only accessible through this partner. In contrast, if ego’s social relations consist of many opportunities to span structural holes, the loss of one connection is marginal, because ego still has other brokering opportunities [5].

### Gender differences in professional networks

Apart from highlighting the general effects of different network structures, extant network studies have revealed significant differences in the professional networks of males and females [1, 32, 33]. Burt [1] has shown that there are differences between the networks of successful men and successful women with respect to structural holes: men benefit more from networks that afford them the chance to occupy many structural holes, while women benefit more from the inferior and more risky networks that contain few structural holes. In such networks, women rely on the support of sponsors in order to benefit from the structural holes. ‘Women supposedly have to borrow social capital from sponsoring, strategic (read male) network partners [1] to be as effective in their careers as their male counterparts’ [34]. To explain why women benefit from more risky networks, Burt [1] concluded that women have less legitimacy within an organisation and therefore need a different network configuration compared to their male colleagues in order to achieve success.

However, Burt did not elaborate on the reasons for this legitimacy problem. The theory of social identity [35] can help to understand this problem. The theory predicts that individuals develop a positive self-image by comparing their own group to other groups. Gender is one aspect of an individual’s social identity [36]. Many authors whose work are grounded in social identity theory have found that women have a lower status than men, because men traditionally occupy high-level positions [36, 37]. According to these works, in order to improve their position, low-status members (i.e., women) typically prefer to identify with a higher status out-group member (i.e., men) and find interactions with female in-group members less attractive [36]. As a result, women identify with those of their peers who belong to a high-status work group and not with other women [38]. This behaviour influences the development of women’s professional networks: women who work and connect with close peers may come to occupy few structural holes, but are likely to lend benefits from a close peer, who is functioning as sponsor. This suggests that the gender-related differences, described further up, can be attributed to the attempts of women to improve their status or increase their legitimacy by using sponsors to gain benefits.

### Numeric representation and tokenism theory

Although the legitimacy deficit, that women have to cope with, explains the differences in the network structures that the two genders most benefit from, the causes of this deficit have yet to be addressed in the network literature. Drawing on tokenism theory [11], we suggest that within organisations, the legitimacy deficit is related to the proportion of women among staff. Kanter [19] distinguished between different minority situations: she described minorities between 15% and 40% of a population as tilted groups and minorities of less than 15% as skewed groups, whose members she called tokens.

Kanter and other researchers have identified three main disadvantages that result from being a token: first, tokens are more visible to their direct peers than the rest of the group and for that reason under more performance pressure [19, 39]. Second, the majority group can easily exaggerate the differences between itself and the skewed group and thus isolate the latter [19]. By isolating women, the dominant majority prevents them from gaining equal access to elite or important networks [2, 3, 32]. Third, tokens are associated with assimilation or role encapsulation. This means that the dominant group has specific distorted expectations of how tokens behave and of the abilities they possess [11].

Although several empirical studies have confirmed the negative effects of token status, the theory has received some criticism questioning the negative effects (e.g., [40–42]). Discussing this criticism at length is beyond the scope of this paper; instead, we refer to the recent review of Watkins et al. [12]. Their review covers 80 empirical research papers on tokens, published between 1991 and 2016. The authors can show that the notion of tokens is still relevant today and worth a deeper investigation.

Kanter described her tokenism theory as gender-neutral. The disadvantages that result from being a token were assumed to apply equally to women and men. However, later studies found that while women are typically affected negatively by token positions, men are not [43–45]. These gender differences can be attributed to different expectations. Sharing the characteristics of people who have already achieved success signals superiority [39]. Men, who have traditionally dominated the top ranks in most organisations, are ascribed as better qualified and more suitable for senior positions than women [46–48]. According to the proven success model, decision-makers prefer candidates who are similar to existing leaders and therefore promote men [49]. As a result, expectations can serve as an explanation for the legitimacy deficits women face: women benefit from a different network configuration than men and should use a network with sponsors, which implies to occupy few structural holes. We expect for the token situation, that Burt’s [1] findings regarding the gender network differences holds true.

#### Hypothesis 1a

The proportion of women in a faculty will moderate the relationship between the number of structural holes and the number of years to promotion, such that the time without promotion will be shorter for women in token positions who occupy few structural holes compared to women in token position who occupy many structural holes.

Although expectations and ascriptions of status are influenced by past experience, there is evidence that attitudes change when the proportion of women in the workforce increases. In turn, this leads to changes in the way individuals are seen and evaluated [50]. Ely [36] suggested that an increase in the proportion of women in higher positions positively influences the status of women because it signals that women are capable of reaching higher positions. Stereotypes are likely to fade when more women are present and information about their true behaviour is pervasive [19].

A number of studies have shown empirically that increasing the ratio of women in the workforce has various effects. An Israeli study by Pazy and Oron [44], based on data from standard appraisals of performance among military officers, showed that the token status of females is linked to negative outcomes: the authors found that women’s performance was rated lower than that of men when women were tokens in their units. This, however, changed when women ceased to be tokens. In the academic context, Maranto and Griffin [51] found that women face a feeling of exclusion, especially when they have a low proportion.

We expect that an increase of women influences the outcomes of networks as well. In a setting where the gender ratio is more balanced, i.e., where the proportion of females is above 15%, women have more legitimacy and do not need a sponsor for having success with a specific network. It allows women to network in the same way as men and to be successful with a network in which they occupy many structural holes. This is contrary to what Burt [1] suggested, but is in line with other researchers, who found that gender does not seem to influence the way in which individuals benefit from their networks (e.g., [9, 52]). By using the tokenism approach, we can explain these contrary findings. To put our conclusions in more formal terms, we expect that the proportion of women on advanced levels moderates the effect of structural holes on the promotion speed.

#### Hypothesis 1b

The proportion of women in a faculty will moderate the relationship between the number of structural holes and the number of years to promotion, such that the time without promotion will be shorter for women in non-token positions who occupy many structural holes compared to women in non-token position who occupy few structural holes.

Further, these arguments can help to answer the more general question whether women should have a different network structure than men. For example, Burt [1] has suggested this. We expect that the network structure of successful women is the same as for successful men, in a situation in which women are not tokens.

#### Hypothesis 2

When women are in non-token positions, the effect of occupying many as compared to few structural holes on their number of years without promotion is identical to the effect of these two network configurations on the promotion speed of men.

## Method

### Sample

Our analysis relies on a full-scale longitudinal sample of 844 researchers at a Swiss university who had reached one of the following ranks in 2013: assistant professor, associate professor, full professor and ‘titular professor’. The latter is an honorary title that carries teaching duties, but has no claim on a chair. We chose this particular university because it is one of the biggest and most diversified universities (i.e., with the broadest range of disciplines) in Switzerland. In our sample, we included only staff eligible for one of the professorial titles listed above. We decided to focus on professors, rather than all employees, because in the higher ranks women are still under-represented and more likely to be affected by tokenism than in the lower ranks [53]. The data set covers the period 2008–13, with annual observations. We consulted a number of different sources to create a unique data set that suited our purposes. All information used is either available to the public or at least to all members of this particular university and has been collected by hand. Whenever possible, to create our variables we used official databases and lists (such as lists of courses, committee members, etc.). When this was not possible, we used the professors’ publicly available CVs.

A major advantage of analysing network data from universities is that universities comprise faculties that work independently of each other. The university we chose consists of seven faculties that are subdivided into 168 departments. On average there are 4.7 professors per department; however, the departments vary strongly in size: in some, there is only one professor, while the largest department numbers 75 professors. Because of this variation in size of departments, we did not include departments as fixed-effects in the main regression analysis. (However, we did this as robustness check, please see S1 Table.).

The proportion of female professors varies strongly among the seven faculties, ranging from 5.8% to 34.8%. During the sample period, the university employed 844 professors, 22.9% of whom were females. In the entire sample (all years) 39.9% of the professors had reached the highest possible hierarchical level, i.e., they were tenured (employed) as full professors. However, they did not have the ‘full professor’ rank at the beginning of our observation. Further, 15.4% were associate professors (also tenured), 8.7% were assistant professors (mostly untenured) and 27.5% were titular professors with notable appointment (mostly tenured). The remaining 8.4% were scholars who became appointed to a professorial position by 2013, but did not have this position in the first observation(s).

In general, academic staff has the chance to be internally promoted from the position of assistant professor or senior lecturer to that of associate professor and eventually to a full professorship. In Switzerland, the academic career works differently compared to other countries, such as UK or USA [21]. Getting an internal promotion is not taken for granted and there is a tough internal competition for reaching the goal of a full professorship [22]. In the Swiss system, which is influenced by the German tradition, the full professor is ‘perceived as the only true member of the profession, while other ranks represent assistantships and apprentices for these positions’ [22]. In comparison, the Anglo-Saxon tradition is less exclusive and hierarchical [22]. Additionally, in Switzerland, there exists only a bunch of universities, so outside options within Switzerland are limited. Researchers tend to stay at one university, resulting in a long internal career [20]. Therefore, our university can function as an internal labour market, similar as it is the case in other academic systems in Europe [20].

In our case university, performance (indicated, for example, by the publication record) is an important factor in achieving internal promotion. However, there is a difference regarding the importance of the publication performance for receiving a promotion at all and for the speed of receiving a promotion. For the speed of promotion, the performance is less important. This has also been found by other researchers [10, 54]. For the speed of promotion, having supporters high up in the hierarchy and the backing of the department, the faculty and the university management is a definite advantage. As in other European countries, networks play an important role in the advancement of academic careers in Switzerland [55]. A single person does not decide who is getting a promotion, but a group of people on different hierarchical levels does. In our sample, we concentrate only on individuals who are eligible to receive a promotion into or in the professorial ranks. For those reasons, our sample is highly suitable for testing how structural holes affect internal promotion and whether there are differences that can be related to the gender of candidates and the proportion of women in a faculty.

### Social network data

To analyse the professional networks, we measure the embeddedness of the professors in the context of the university as a whole by matching four types of data on objective affiliations. We assumed a tie between two individuals, when we found at least one relation in any of the four types of affiliations. This is an innovative approach for measuring social network in academia, because most studies use rather simple network measure such as membership [10] or rely on co-authorship alone [14, 52, 56, 57]. Therefore, we reduce shortcomings of earlier publications, by including otherwise unobserved ties [57].

The first approach to capture ties between researchers is based on co-authorship. This approach is a very common approach and is often used by studies examining the social networks of researchers (e.g., [52, 57]). To collect information on the publication records of all researchers, we used a publicly accessible central university database. Because of reporting regulations, all researchers at our sample university are obliged to list all of their publications including co-authors in this central database and to update this list once a year. We used co-authors, as a network tie, regardless of their university affiliation or hierarchical position. In total, we identified 86,114 authors who functioned as network ties.

Second, we identified networks based on teaching collaborations. At the sample university, all professors have very similar teaching duties and are free to collaborate on a course or seminar. We think this is an innovative and promising approach to visualise the social networks of scholars. We collected data for every semester from the official course listings. In total, we used 19,334 course units taught by at least two lecturers each to create network ties.

The third type of network ties reflects membership in university committees. Drawing on an official university database containing information on the university’s 54 committees and their members, we measured each professor’s committee membership. Committees connect all faculties and are responsible for specific areas, such as student affairs. The committees are open to candidates among pre-tenure academic staff and university regulations demand that they comprise representatives of all ranks. Depending on the size and importance of the committee, even students and postdocs can be elected as representatives. Women are not especially encouraged to join committees just because of their gender. The proportion of women in committees (21.88%) is nearly the same as in the overall sample (22.9%), which indicates that women are neither over- nor under-represented. We assumed that all members of one committee know each other. Committees consist on average of eight members with a maximum of 19 members. Several meetings take place per year. We conducted several interviews with the members of committees. The aim was to verify that members of the same committee know each other. We found that, members know all the other committee members and therefore created ties to them. Overall, 321 university members are affiliated with at least one committee.

Similarly, the fourth type of network ties is based on the membership in competence centres. Competence centres are research groups for specific areas, such as human rights. Like committees, these centres connect different faculties and departments. We measured each professor’s membership of every competence centre to which he or she belonged. Each such network includes the focal professor and the other members of that centre. The respective competence centre mostly puts forward new members, which implies that the candidates’ interests have to be well known and that membership is the result of informal ties. Similar to committees, we conducted some short interviews with members of competence centres, in order to verify that members of one competence centre know each other. Again, it turned out, that this is the case. We collected our data from the webpage of each competence centre, which includes a list of members. However, we were not able to collect data on past members and on the joining date. We therefore only include ties generated from membership data for 2013. To check the validity of this procedure, we compared the network information we had for 2013 with that for 2012. Although the results were marginally different, we did not find significant differences. In the entire sample, 417 professors are members of one to four competence centres.).

### Dependent variable

#### Years without internal promotion

Our dependent variable measures the career advancement of researchers (excluding full professors, once they have reached this status). In line with similar studies (e.g., [10, 58]), to measure career success in academia we looked at the speed of internal promotion. We counted the number of years that individuals who were eligible for promotion had spent in their current position without being promoted. Higher values indicate slower career progress. To generate this variable, we used official data on each researcher’s career. In our first observation year, we already know how long an individual has been in this position and how long she or he is eligible to be promoted.

### Independent variables

#### Structural holes

To measure a professor’s success in a network, we used the number of structural holes that an individual occupied, using Burt’s constraint measure C [5]. This measure accounts for the size, density and hierarchical structure of a network. Using the number of structural holes to derive this measure, which is the reverse indicator of constraint (1-C), enabled us to simplify the interpretation of our results. Constraint reflects the extent to which ego’s network partners are connected to one another. Lower values of constraint imply that it is more likely for an individual to bridge structural holes [59]. Higher values of constraint indicate that it is less likely for an individual to bridge structural holes. Instead, successful individuals use sponsors and lend the advantages of structural holes from them [1]. Please note that the measure for structural holes is a continuous measure. However, we expect certain effects for low and high values. This is in line with the work of Burt [5] and other researchers (e.g., [15]) who have used the concept of structural holes. For further details about structural holes and how the measure is derived, see Burt [5].

#### Female

We measured the gender of a professor as a binary variable (0 = males, 1 = females).

#### Proportion of females

Based on the official statistics provided by the university, we coded the proportion of female professors in a faculty in every year (excluding lower ranking employees, such as clerical staff and postdocs). In the dataset, 60% of all females are women in token positions, meaning they form a subgroup smaller than 15% [19].

### Control variables

#### Publication index

We use a publication index as a control for performance. It is not easy to compare the publication performance of academics in different disciplines and at different stages in their career. Typical citation metrics, such as Hirsch’s h-index [60] or Egghe’s g‐index [61] deliver biased results in such cases. The metric we used to measure publication performance is the hI, annual index. This is a suitable measurement to compare researchers at different career stages and from different disciplines. For example, it includes discipline-specific controls, such as the number of co-authors. For more information how this index is created and why it is suitable for interdisciplinary comparisons, please see the paper of Harzing et al. [62]. To generate this index we used the software Publish or Perish 4, which processes data retrieved by Google Scholar.

#### Signalling talent

Signalling talent, i.e., credibly conveying attractive information about oneself to another party, is important in job markets and for promotion [63, 64]. In academia, the university where a professor has been educated or was previously employed plays an important role in his or her quality assessment quality assessment. A previous affiliation with a highly respected university, such as Harvard or Stanford, for example, conveys credibly positive information about a person’s abilities to his or her peers and to prospective employers and improves the outcome of a quality assessment. This control has the aim to rule out that a positive signal about the abilities of a person instead of the network is responsible for the speed of promotions. In our case study, we recorded the university where the professors had received their PhD or had gained a postdoctoral qualification, both of which are prerequisites for a professorship in many European countries. When a university was included either in the Top 200 of the QS World University Ranking 2012 or in the Top 200 of the Times Higher Education Ranking 2012–13 we coded this university with 1 and if it was included in both lists with 2. We then calculated the mean of the number of universities an individual had attended up to the point of our observation. For example, a professor who had completed his or her PhD and gained a postdoctoral qualification at universities included in both lists was assigned ‘2’, which represents the highest degree of signalling. In contrast, someone formerly employed at three different universities of which only one is included in either of these lists would receive a much lower ‘1/3’. To generate this and the next two control variables we coded the CV of each professor.

#### Editor and board positions (log)

Professional scientific journals are the primary publication outlets of research communities. The editorial boards of these journals play a considerable role both in the dissemination of information and in its evaluation by an expert audience. Their members tend to be regarded as experts in their field [65].

Being appointed to an editorial board is not only a great honour, but can also be seen as an indicator of scientific quality. Gibbons and Fish [66] confirm this idea: ‘Certainly, the more editorial boards an economist is on, the more prestigious the economist.’ Consequently, serving on an editorial board can be regarded as indicative of scholarly quality among one’s peers [67]. In our sample, we counted the number of positions that a professor held as editor or board member of an academic journal.

#### Different organisations (no.)

We counted the number of universities at which an individual had been employed before taking up the position at our sample university. This control can be seen as a measure of academic experience and has already been used in studies on networks and career success (e.g., [27]). We included all universities at which an individual had been employed for at least 6 months after the completion of his or her PhD.

#### Committee memberships (no.)

This variable reflects the number of committees on which an individual serves as a member; in other words, a measure for the size of the individual network. Given that larger networks offer more opportunities to occupy structural holes, but also require more time and effort for networking, we used this variable to control whether individuals benefit from the structure of their network or merely from having a larger network.

#### Competence centre memberships (no.)

This variable reflects the number of competence centres which an individual is a member of. Again, we used this variable to distinguish between the effects of a network’s structure and of a network’s size.

#### Department size

This variable measures the number of an individual’s colleagues with a professorial title in the same department. Larger departments might offer more opportunities for networking, because the members of the same department are more likely to work together and might thus benefit from building a network. At the same time, larger departments may mean a higher constraint and thus fewer opportunities to bridge structural holes.

#### Network size

This variable represents the number of actors that an individual is directly connected to. This variable represents all potential sources of contact; namely, co-authorship, collaborations, committee membership and membership of a competence centre.

The choice of these control variables is in line with previous studies in an academic setting (e.g., [17, 52]).

In addition to the control variables, we included fixed-effect dummies in our regression models to control for year, faculties and professorial ranks. In Table 1 means, standard deviations and correlations of our variables are shown.

**Table 1:** Means, standard deviations and correlations.

	Variable	Obs	Mean	Std. Dev	Min	Max	1	2	3	4	5	6	7	8	9	10	11
1	Years without internal promotion	2099	4.258	4.149	0	31	1										
2	Publication index	2099	.812	.596	0	3.14	-.165	1									
3	Signalling talent	2099	1.029	.875	0	2	-.043	-.044	1								
4	Editor/board (log)	2099	-.230	.523	-.43	2.27	-.084	.083	.063	1							
5	Different orgas. (no.)	2099	.700	.821	0	4	-.201	.084	.011	.167	1						
6	Committee member. (no.)	2099	.078	.311	0	3	.062	-.060	-.036	.001	.044	1					
7	Competence member. (no.)	2099	.744	.943	0	4	-.108	.278	-.048	.075	.140	.105	1				
8	Department size	2099	13.474	18.106	1	75	.152	-.345	.042	.022	.012	-.009	-.112	1			
9	Female	2099	.223	.420	0	1	-.089	-.114	.033	.024	.056	-.001	-.083	-.056	1		
10	Proportion of females	2099	.150	.095	.06	.35	.060	-.492	.021	.067	.188	.025	-.082	.360	.096	1	
11	Network size	2099	83.067	90.226	0	593	-.035	.509	.040	-.066	-.114	.018	.233	-.295	-.145	-.452	1
12	Structural holes	2099	.888	.1637	0	1	-.060	.256	-.018	-.061	-.048	.043	.142	-.225	-.021	-.344	.339

## Analysis

To test our hypotheses, we used the set of longitudinal data covering the period 2008–13 with ‘years without internal promotion’ as the dependent variable (Tables 2 & 3). We used a time lag of one year for the network based on co-authored papers. We expect that authors collaborate on a joint work for at least one year before publication. We constructed random effect models and negative binomial models.

**Table 2:** Longitudinal model predicting ‘years without internal promotions’ for women only.

	Model 1a Random effect	Model 1b Random effect	Model 1c Negative binomial
Publication Index	0.23	0.05	0.01
	(0.51)	(0.66)	(0.18)
Signalling talent	-0.35	-0.23	-0.01
	(0.32)	(0.35)	(0.09)
Editor/Board (log)	0.80+	0.81+	0.24+
	(0.46)	(0.48)	(0.13)
Different org. (no.)	-0.47	-0.30	-0.11
	(0.29)	(0.36)	(0.10)
Committee member (no.)	0.68	1.63*	0.32
	(0.77)	(0.79)	(0.23)
Competence member (no.)	-0.31	0.16	0.07
	(0.33)	(0.36)	(0.11)
Department size	-0.02	-0.03	-0.00
	(0.02)	(0.04)	(0.01)
Network size		0.00	0.00
		(0.00)	(0.00)
Structural holes		8.16**	2.75**
		(2.64)	(0.92)
Proportion of females		21.11+	7.79*
		(10.88)	(3.49)
Proportion × struct. holes		-27.22**	-9.02**
		(8.73)	(2.95)
Constant	4.61***	-0.27	2.78
	(0.72)	(3.83)	(2.81)
Year fixed-effects	No	Included	Included
Faculty fixed-effects	No	Included	Included
Professorial fixed-effects	No	Included	Included
R-sqr	0.04	0.10	
Wald-Chi2	5.7	91.25***	83.52***
N	481	481	481
N-groups	115	115	115

Prediction of the dependent variable ‘years without internal promotions’ including only females and using the proportion of females as a metric variable. Standard errors are in parenthesis.

^+^< p. 0.10;

*< p 0.05;

**< p 0.01;

***< p 0.001.

**Table 3:** Longitudinal model predicting ‘years without internal promotions’ by token-split.

	Model 2a Random effect	Model 2b Random effect	Model 2c Negative binomial	Model 2d Random effect	Model 2e Random effect	Model 2f Negative binomial
	faculty with token women (< = 15%)			faculty with non-token women (>15%)		
Publication index	-0.78*	-1.20**	-0.32***	-1.20+	-1.62*	-0.47**
	(0.37)	(0.37)	(0.09)	(0.71)	(0.72)	(0.15)
Signalling talent	0.00	-0.06	0.03	-0.47	-0.48	-0.08
	(0.21)	(0.21)	(0.05)	(0.32)	(0.33)	(0.07)
Editor/board (log)	0.50	0.67+	0.22*	-0.41	-0.46	-0.08
	(0.38)	(0.39)	(0.09)	(0.49)	(0.50)	(0.11)
Different org. (no.)	-0.53*	-0.35	-0.11+	-1.07***	-0.99**	-0.24***
	(0.26)	(0.26)	(0.06)	(0.32)	(0.33)	(0.06)
Committee member (no.)	2.01***	1.75***	0.42***	0.79	0.58	0.16
	(0.46)	(0.45)	(0.12)	(0.49)	(0.49)	(0.12)
Competence member (no.)	0.20	0.13	0.05	-0.55+	-0.67*	-0.13+
	(0.21)	(0.21)	(0.05)	(0.33)	(0.34)	(0.07)
Department size	-0.01	0.00	0.00	0.01	0.01	0.00
	(0.03)	(0.03)	(0.01)	(0.01)	(0.01)	(0.00)
Female		-4.84*	-1.74*		-0.59	-0.00
		(2.00)	(0.68)		(0.90)	(0.24)
Network size		0.01***	0.00***		0.01***	0.00**
		(0.00)	(0.00)		(0.00)	(0.00)
Structural holes		-0.82	-0.12		-1.52***	-0.21*
		(0.97)	(0.27)		(0.44)	(0.11)
Female × struct. holes		5.19*	1.89**		0.18	-0.02
		(2.12)	(0.73)		(0.78)	(0.23)
Constants	5.31***	5.59***	17.24	6.70***	8.04***	15.98
	(0.58)	(1.06)	(209.73)	(0.79)	(0.88)	(655.19)
Year fixed-effects	No	Included	Included	No	Included	Included
Faculty fixed-effects	No	Included	Included	No	Included	Included
Professorial fixed-effects	No	Included	Included	No	Included	Included
R-sqr	0.06	0.07		0.18	0.19	
Wald-Chi2	121.23***	193.26***	159.26***	137.66***	169.17***	139.17***
N	1363	1363	1363	736	736	736
N-groups	347	347	347	245	245	245

Prediction of ‘years without internal promotions’ with both genders and by splitting for faculties with token and non-token females. Standard errors are in parenthesis.

^+^< p. 0.10;

*< p 0.05;

**< p 0.01;

***< p 0.001.

In order to verify the validity of our main models, we did some additional regression models. The results of these models are in the Supporting Information files. S1 Table is a replication of the models 2b and 2e in Table 3, with departments instead of the faculties as fixed-effect controls. We clustered very small departments into the reference category. Otherwise, there are several problems, such as multicollinearity.

S2 Table is a replication of the models 2b and 2e in Table 3, as well. Here we separated for the professorial ranks. We did this for the two ranks that include most promotions. For the other ranks, we do not have enough observations to specify valid models.

S3 Table shows the results of a simple logit regression. The aim of this model is to show the importance of publication performance for receiving a promotion at all. In our main models, the publication index is not always significant. As explained in the method section, the publication performance is not that important for the speed of promotion. Therefore, these models aim to emphasize that there is a difference between receiving a promotion at all and the speed of receiving a promotion.

## Empirical findings

Table 2 contains the empirical results of the regression in which we included only females. In model 1a we used only the control variables. The results show that no variable has a significant effect on the promotion speed of female professors. Model 1b and 1c includes the network variables and the fixed-effects. Here the number of structural holes (p<0.01) have a significant effect on the promotion speed of female academics. The interaction effect between the number of structural holes and the proportion of females within a faculty (p<0.01) is also significant.

**Fig 1:**
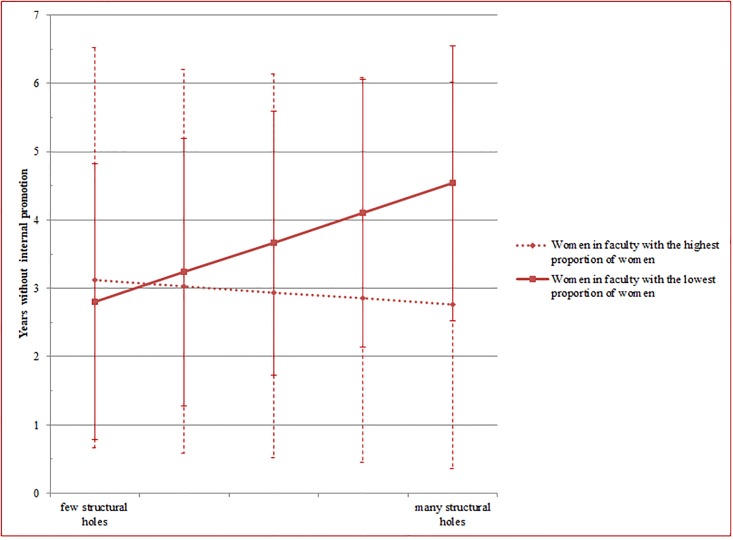
Illustration of the longitudinal model for women only (Table 2, Model 1b, including 95% confidence intervals).

Fig 1 illustrates the results of this analysis. Both lines represent the situation in the faculties with the highest and the lowest proportion of women. In line with Hypothesis 1a, the findings show that women in token positions who occupy many structural holes are promoted more slowly than women who occupy few structural holes. The findings also confirm Hypothesis 1b: On average women in non-token positions who occupy many structural holes are promoted nearly three months earlier than women who occupy few structural holes.

The regression results to test Hypothesis 2 and to validate Hypotheses 1a and 1b are shown in Table 3. Models 2a, 2b and 2c correspond to faculties in which women are in token situations, while models 2d, 2e and 2f correspond to faculties in which women are in non-token situations. In line with Kanter [19] and most authors who draw on tokenism theory (e.g., [68]), we used 15% as the threshold for the proportion of female professors in a faculty. In models 2a and 2d we included only the control variables. In Model 2b and 2c (token situation) the interaction effect between female and structural holes is positive and significant (at least on a p<0.05 level), which lends further support to Hypothesis 1a and validates the results in Table 2. This finding indicates that in token situations women and men benefit from differently structured networks.

**Fig 2:**
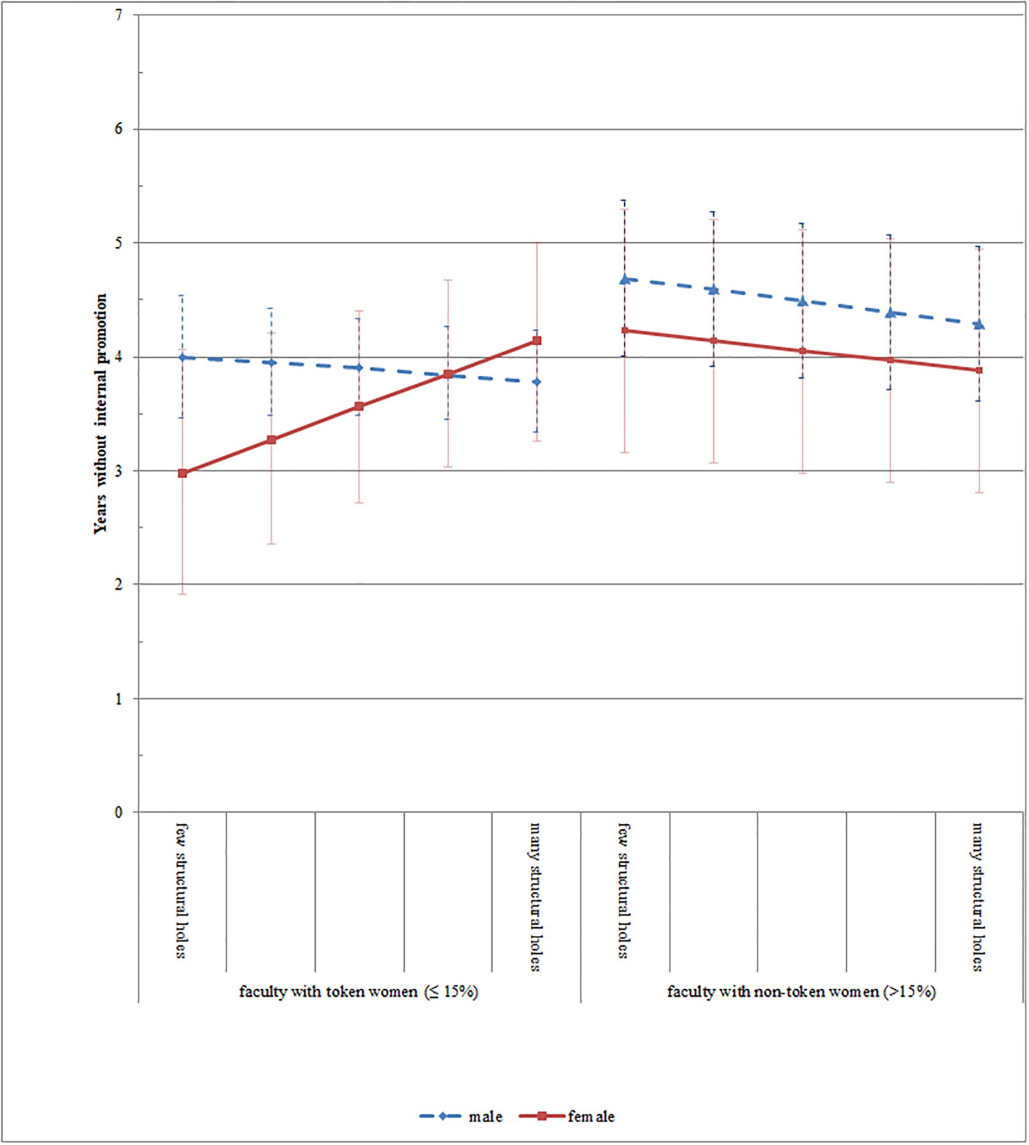
Illustration of the longitudinal model based on both genders and by splitting the sample in faculties with women in token and non-token positions (Table 3, Model 2b and Model 2e, including 95% confidence intervals).

In Model 2e and 2f the interaction effect between females and structural holes is not significant. This means that in non-token situations, women and men benefit from identically structured networks. Further, in Model 2e and 2f the ‘structural hole’ coefficient is significantly negative (at least on a p<0.05 level), which implies that both men and women benefit from occupying structural holes in the non-token situation. Again, this validates the findings in Table 2 and lends further support for Hypothesis 1b. In Fig 2, these results are graphically illustrated. The figure on the left shows that women in token situations can decrease their time to promotion by about one year, by occupying only few structural holes. In contrast, women in non-token situations can decrease their time to promotion by about four months, by occupying many instead of few structural holes. Independently of the gender proportion, men are promoted between two and three months faster if they occupy many structural holes.

The lines for men and women on the right side of Fig 2 illustrate that in non-token situations network effects are gender independent. This supports Hypothesis 2 that women and men benefit from identically structured networks in non-token situations. In the regression table in Model 2e and 2f, the non-significant interaction effect between females and structural holes illustrates this.

Regarding the control variables, the findings show that scholars in non-token situations benefit from having experience at different universities. The variable ‘network size’ is highly significant and positive (p<0.001), which shows that building larger networks is not a successful strategy and at the same time supports our assumption that the right network configuration is essential for success. A strong publication performance is also important.

## Discussion and conclusion

Our paper combined social network theory with tokenism to examine the relation between gender and career progress. The results of our analysis suggest that gender influences the way in which the structural features of an individual’s network affect his or her career prospects. More precisely, we found that when women are in token situations, i.e., when the proportion of women is below 15%, they benefit from networks with few structural holes; while their male colleagues benefit from networks with many structural holes. This result is in line with Burt, who reported that men and women benefit from different network structures when it comes to career success [1]. We concluded that these differences are attributed to differences in status and in expectations: when women are tokens at their workplace, their proportion is too low to challenge negative expectations, status and stereotypes [19, 36]. In contrast, we found that in non-token situations, i.e., when the proportion of women exceeds 15%, both men and women benefit from the same network structures. Over this threshold, it appears that a minority becomes too numerous to be isolated by the majority and too visible to be ignored by third parties. When the proportion of women increases, third parties have more opportunities to observe female behaviour, which in turn refutes stereotypes and enables women to overcome problems of status. As our results show, when women are not in a token position they can benefit from the same type of networks as their male colleagues with regard to career success. In such settings, women do not need to use sponsors, as suggested by Burt [1], in order to benefit from structural holes, because they enjoy status and legitimacy which allows them to occupy and benefit from structural holes directly.

This finding refutes the claim that women generally benefit from different network structures, as Burt [1] had argued. It also explains why more recent studies have found no evidence for gender-specific network effects (e.g., [9, 52, 69]). Moreover, in contrast to Burt [1], we observed that women benefit from networks with many structural holes. This principle might apply to other settings, such as the film industry, where Lutter [70] found that networks associated with lower chances to occupy structural holes hinder the careers of females. Overall, our findings help explain some of the apparent inconsistencies in the literature in two ways: First, they highlight the influence of gender proportions on network effects. Second, they provide an answer to authors [7] who have called into question Burt’s [1] findings on the network strategies which women benefit from.

Our results also contribute to the debate about female preferences with regard to network structure. Some researchers argue that women and men generally prefer different network structures and that these gender-related differences are inherent [6, 8, 24], while others insist that the main determinant is the situation or the environment (i.e., differential access to opportunity of men and women) and not gender per se [2, 24, 25]. Our study provides evidence for the latter argument and shows that within an organisation the proportion of women on advanced levels is an important situational factor that might influence how individuals configure their networks. In a token situation, women should use networks that differ in their structure compared to the networks men use. However, this does not imply that women generally prefer a different structure. As we pointed out, when the proportion of women is high, they benefit from the same network structure as men. As a result, former studies arguing that women prefer different structures might come to this conclusion because women chose a different structure than men. However, women act this way in order to be successful and not because women have a different preference in general.

Finally, we contribute to the literature on the benefits of individual networks in academic contexts: We show that networks are an important determinant of the speed which academics are promoted with. Previous studies only examined how networks influence publication performance [14] or the probability of reaching a particular step on the career ladder [17, 71, 72], but hardly any study used network analysis combined with career speed.

In our discussion of tokens versus non-tokens, we argued that the tipping point, at which a minority cease to have token status, lies around 15%. While this threshold seems appropriate in the context of the Swiss academic settings, in other settings other thresholds might be more appropriate. For example, there is evidence that in a corporate board setting the presence of at least three women suffices to create positive effects on firm performance [73] or on the level of firm innovation [74]. Acknowledging that studies that are grounded in other theories propose higher tipping points, we included a metric proportion measure in the first of the two analyses (Table 2).

### Limitations and directions for future research

Like all studies, this one has certain limitations. First, our university data set is somewhat idiosyncratic and, therefore, the findings may not be fully transferable to other types of organisations, such as private-sector companies. For example, as we discussed further up, the threshold at which women benefit from structural holes might be lower or higher in other organisational forms. Further, we only use data of a single university, which limits the generalizability of the study.

Second, we do not measure the quality of ties. For example, we do not differentiate between weak and strong ties like Granovetter [75] did. There might be hidden effects due to the quality of a tie. However, we think that it is not possible to integrate every perspective and approach in one study.

Third, we used objective data, such as data based on network affiliations and instead of data based on questionnaires. On the one hand, this choice has certain disadvantages. One problem with our type of data is that it provides no direct information about gender differences in behaviour at the workplace. Further, it provides no information regarding negotiations with colleagues and employers. Both of these factors might be important. For example, self-confidence might have an influence on the impact of token status [76]. On the other hand, using objective data is an advantage, because questionnaire data is often affected by a subjective bias. The use of objective data also allowed us to avoid the problem of non-random sampling that most studies on networks suffer from [77]. Several studies on social networks rely on objective data [30]–an approach that has proven to be useful.

Despite their drawbacks, these limitations also can be viewed as potential avenues for future research: First, it would be interesting to see whether other studies can replicate our results by using other types of data sources drawn from other organisations, other tools, such as ego-centric questionnaires, or models with a broader range of control variables. For example, factors such as career breaks or self-confidence could help explain gender differences in career patterns in greater detail. Second, future studies could examine how different ratios of women to men relate to absolute group size and how these ratios interact with the career of men and women.

## Supporting information

S1 Table Longitudinal GLS-model with random effects predicting ‘years without internal promotions’ by token-split, with departments as fixed-effects. Prediction of ‘years without internal promotions’ by splitting in token and non-token females and using department instead of faculty as fixed-effects. Standard errors are in parenthesis. +< p. 0.10; *< p 0.05; **< p 0.01; ***< p 0.001.
(DOCX)

S2 Table Longitudinal GLS-model with random effects predicting ‘years without internal promotions’ by token-split, separated for the professorial ranks. Prediction of ‘years without internal promotions’ by splitting in token and non-token females and using only two professorial ranks. Standard errors are in parenthesis. +< p. 0.10; *< p 0.05; **< p 0.01; ***< p 0.001.
(DOCX)

S3 Table Longitudinal Logit-model predicting ‘received promotion’. Prediction of ‘received a promotion’ without any gender or network variables. Standard errors are in parenthesis. +< p. 0.10; *< p 0.05; **< p 0.01; ***< p 0.001.
(DOCX)

S1 Fig Ego occupies a structural hole and thus acts as a broker between two groups. Without ego, the group on the left would be disconnected from the group on the right.
(TIFF)

S2 Fig Ego’s network, in which ego ‘lends’ the advantages of structural holes from a sponsor. (TIFF)
