# Supplementary material for: The influence of gender ratios on academic careers: Combining social networks with tokenism
Source: PLoS One. 2018 Nov 16;13(11):e0207337. doi: 10.1371/journal.pone.0207337 (PMC6239321; doi:10.1371/journal.pone.0207337)
Supplement: S1 Table — Prediction of ‘years without internal promotions’ by splitting in token and non-token females and using department instead of faculty as fixed-effects. Standard errors are in parenthesis. +< p. 0.10; *< p 0.05; **< p 0.01; ***< p 0.001. (DOCX) [file pone.0207337.s001.docx]

**S1 Table. Longitudinal GLS-model with random effects predicting ‘years without internal promotions’ by token-split, with departments as fixed-effects.**

|  | Token women (<=15%) | Non-Token women (<15%) |
| --- | --- | --- |
|  |  |  |
| Publication index | -1.42** | -0.93 |
|  | (0.52) | (1.03) |
| Signalling talent | -0.16 | -0.88* |
|  | (0.29) | (0.41) |
| Editor/board (log) | 0.67 | -0.57 |
|  | (0.53) | (0.57) |
| Different orgas. (no.) | -0.44 | -1.37*** |
|  | (0.36) | (0.38) |
| Committee member. (no.) | 1.99*** | 0.46 |
|  | (0.49) | (0.52) |
| Competence member. (no.) | -0.07 | -0.67+ |
|  | (0.29) | (0.40) |
| Female | -4.37* | 0.20 |
|  | (2.09) | (0.98) |
| Network size | 0.01*** | 0.01*** |
|  | (0.00) | (0.00) |
| Structural holes | -0.83 | -1.58*** |
|  | (0.99) | (0.44) |
| Female × struct. holes | 5.28* | 0.10 |
|  | (2.16) | (0.79) |
| Constant | 9.49* | 9.55*** |
|  | (3.74) | (1.02) |
|  |  |  |
| Year fixed-effects | Included | Included |
| Department fixed-effects | Included | Included |
| Professorial fixed-effects | Included | Included |
|  |  |  |
| R-sqr | 0.32 | 0.33 |
| F-value | 310.14*** | 225.05*** |
| N | 1363 | 736 |
| N-groups | 347 | 245 |

Prediction of ‘years without internal promotions’ by splitting in token and non-token females and using department instead of faculty as fixed-effects. Standard errors are in parenthesis. +< p. 0.10; *< p 0.05; **< p 0.01; ***< p 0.001
