# Supplementary material for: The influence of gender ratios on academic careers: Combining social networks with tokenism
Source: PLoS One. 2018 Nov 16;13(11):e0207337. doi: 10.1371/journal.pone.0207337 (PMC6239321; doi:10.1371/journal.pone.0207337)
Supplement: S2 Table — Prediction of ‘years without internal promotions’ by splitting in token and non-token females and using only two professorial ranks. Standard errors are in parenthesis. +< p. 0.10; *< p 0.05; **< p 0.01; ***< p 0.001. (DOCX) [file pone.0207337.s002.docx]

**S2 Table. Longitudinal GLS-model with random effects predicting ‘years without internal promotions’ by token-split, separated for the professorial ranks.**

|  | Promotions for Associate Professor | Promotions for Associate Professor | Promotions for Titular Professor | Promotions for Titular Professor |
| --- | --- | --- | --- | --- |
|  | faculty with token women (<=15%) | faculty with non-token women (>15%) | faculty with token women (<=15%) | faculty with non-token women (>15%) |
|  |  |  |  |  |
| Publication Index | -1.58** | -1.71+ | -1.00 | -0.57 |
|  | (0.60) | (0.90) | (0.69) | (1.56) |
| Signalling talent | 0.05 | -0.07 | -0.26 | -0.84 |
|  | (0.38) | (0.47) | (0.36) | (0.56) |
| Editor/board (log) | 0.55 | 0.71 | -0.18 | -2.75** |
|  | (0.50) | (0.67) | (0.89) | (1.06) |
| Different org. (no.) | -0.18 | -0.52 | -0.50 | -0.76 |
|  | (0.37) | (0.44) | (0.55) | (0.65) |
| Committee member (no.) | 0.63 | -0.85 | 2.10+ | 1.01 |
|  | (0.47) | (0.58) | (1.14) | (1.02) |
| Competence member (no.) | 0.03 | -0.81+ | -0.08 | -1.97* |
|  | (0.30) | (0.48) | (0.48) | (0.86) |
| Department size | 0.03 | -0.02 | 0.06 | 0.03 |
|  | (0.05) | (0.02) | (0.06) | (0.02) |
| Female | -11.94* | 2.84 | -9.81** | -2.28 |
|  | (5.61) | (2.22) | (3.65) | (1.45) |
| Network size | 0.01*** | 0.03*** | 0.01** | 0.01+ |
|  | (0.00) | (0.00) | (0.00) | (0.01) |
| Structural holes | -1.61 | -2.96** | -3.92+ | -2.07*** |
|  | (2.24) | (0.90) | (2.26) | (0.60) |
| Female × struct. holes | 12.78* | -3.18 | 10.64** | 0.58 |
|  | (6.07) | (2.39) | (3.85) | (1.00) |
| Constant | 5.07* | 7.87*** | 8.15*** | 8.39*** |
|  | (2.31) | (1.47) | (2.31) | (1.48) |
| Year fixed-effects | Included | Included | Included | Included |
| Faculty fixed-effects | Included | Included | Included | Included |
|  |  |  |  |  |
| R-sqr | 0.07 | 0.11 | 0.08 | 0.24 |
| F-value | 84.20*** | 80.58*** | 37.62** | 55.27*** |
| N | 350 | 198 | 596 | 375 |
| N-groups | 110 | 81 | 165 | 114 |

Prediction of ‘years without internal promotions’ by splitting in token and non-token females and using only two professorial ranks. Standard errors are in parenthesis. +< p. 0.10; *< p 0.05; **< p 0.01; ***< p 0.001
