# Supplementary material for: The influence of gender ratios on academic careers: Combining social networks with tokenism
Source: PLoS One. 2018 Nov 16;13(11):e0207337. doi: 10.1371/journal.pone.0207337 (PMC6239321; doi:10.1371/journal.pone.0207337)
Supplement: S3 Table — Prediction of ‘received a promotion’ without any gender or network variables. Standard errors are in parenthesis. +< p. 0.10; *< p 0.05; **< p 0.01; ***< p 0.001. (DOCX) [file pone.0207337.s003.docx]

**S3 Table. Longitudinal Logit-model predicting ‘received promotion’.**

|  | Coef (Stand. Error) |
| --- | --- |
|  |  |
| Publication index | 0.29* |
|  | (0.12) |
| Signalling talent | 0.14+ |
|  | (0.08) |
| Editor/board (log) | 0.09 |
|  | (0.13) |
| Different orgas. (no.) | 0.03 |
|  | (0.09) |
| Constant | -2.53 |
|  | (0.17) |
|  |  |
|  |  |
| Wald chi2 | 10.73* |
| N | 2205 |
| N-groups | 493 |

Prediction of ‘received a promotion’ without any gender or network variables. Standard errors are in parenthesis. +< p. 0.10; *< p 0.05; **< p 0.01; ***< p 0.001
