# Supplementary figures and images for: The influence of gender ratios on academic careers: Combining social networks with tokenism
Source: PLoS One. 2018 Nov 16;13(11):e0207337. doi: 10.1371/journal.pone.0207337 (PMC6239321; doi:10.1371/journal.pone.0207337)

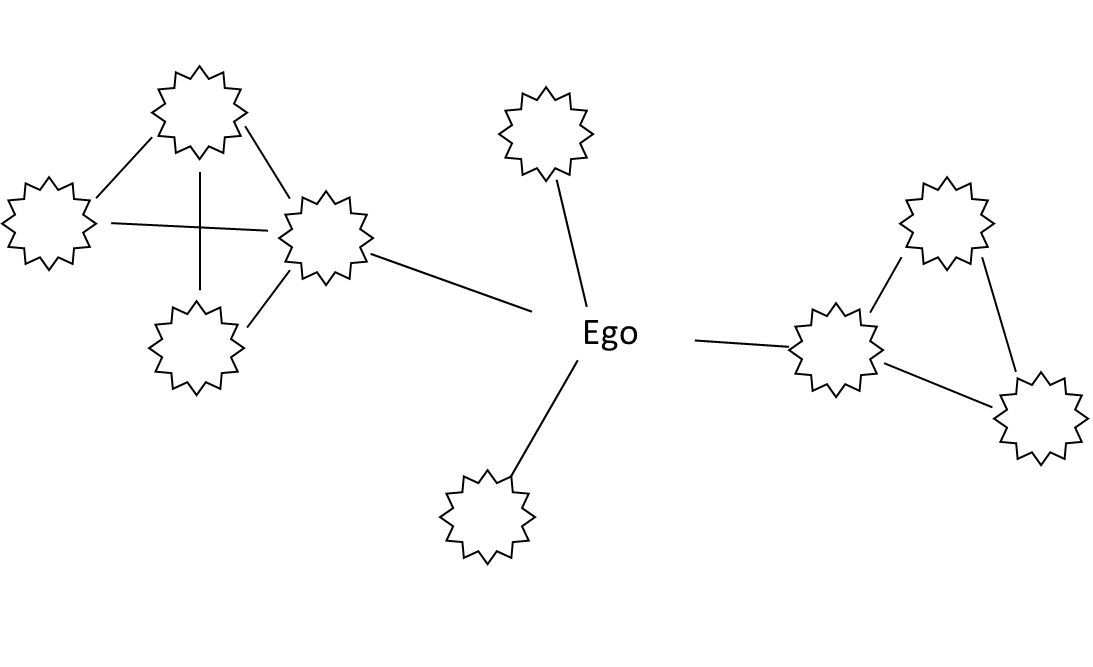

Supplement: S1 Fig — Without ego, the group on the left would be disconnected from the group on the right. (TIFF) [file pone.0207337.s004.tiff]

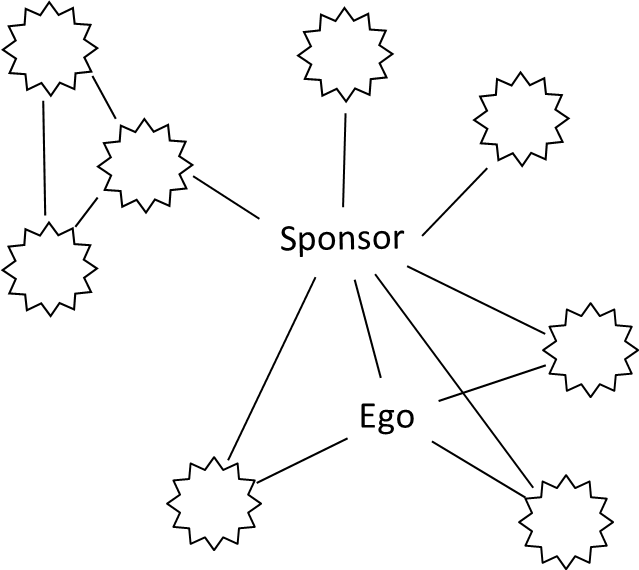

Supplement: S2 Fig — (TIFF) [file pone.0207337.s005.tiff]
